# Sex Differences in Foraging Rats to Naturalistic Aerial Predator Stimuli

**DOI:** 10.1016/j.isci.2019.06.011

**Published:** 2019-06-11

**Authors:** Peter R. Zambetti, Bryan P. Schuessler, Jeansok J. Kim

**Affiliations:** 1 Department of Psychology, Guthrie Hall, University of Washington, Seattle, WA 98195, USA; 2 Program in Neuroscience, University of Washington, Seattle, WA 98195, USA

**Keywords:** Biological Sciences, Animals, Ethology

## Abstract

Rodents in the wild are under nearly constant threat of aerial predation and thus have evolved adaptive innate defensive behaviors, such as freezing or fleeing, in response to a perceived looming threat. Here we employed an ethologically relevant paradigm to study innate fear of aerial predators in male and female rats during a goal-oriented task. Rats foraging for food in a large arena encountered either a 2D or 3D looming stimulus, to which they instinctively fled back to a safe nest. When facing a direct aerial threat, female rats exhibited a greater fear response than males and this divergence maintained when exposed to the environment on subsequent days with no predator interaction, suggesting stronger contextual fear in female rats. These results may have relevance toward exploring neurobiological mechanisms associated with higher diagnosis rates of fear and anxiety-related disorders in women as compared with men.

## Highlights

• Female rats exhibited stronger fear responses to aerial threats than male rats
• A 3D aerial predator was more effective at eliciting fear responses than 2D stimuli
• Contextual fear memories were formed from repeated 3D predator exposures

## Graphical Abstract

**Figure:**
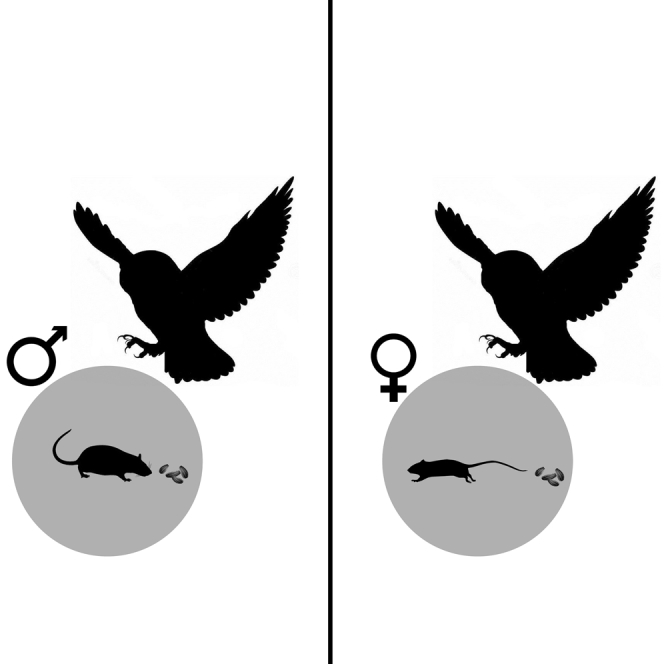

## Introduction

The study of fear in laboratory rats and mice has traditionally utilized paradigms concerned with basic associative fear learning or how fear of specific stimuli are acquired (Kim and Jung, 2018). For example, in Pavlovian (classical) fear conditioning—the most widely used associative fear learning paradigm—an initially neutral conditioned stimulus (CS; e.g., a tone, light, or context) is temporally paired with the presentation of an aversive unconditioned stimulus (US; e.g., an electric shock to the animal's paws), typically over several trials. Through repeated CS-US pairings, the CS becomes a predictor of the US, and its presentation alone elicits fear-related behavior (such as freezing) in the animal. Similarly, in instrumental (operant) fear conditioning paradigms, the focus is on how the animal learns to avoid or terminate an aversive stimulus by responding appropriately to predictive stimuli over the course of several trials. Although these associative paradigms have been useful in studying principles of learned fear and its underlying neural substrates, they address only a facet of fear as a functional behavior intended to keep animals alive in nature (Pellman and Kim, 2016).

In contrast, studies investigating innate (unconditioned) fear employ stimuli that are instinctively threatening to the species being tested. Historically used by ethologists (Kavaliers and Choleris, 2001, Schleidt et al., 2011), innate fear paradigms often use predator cues (Takahashi et al., 2005), or in some cases, actual or simulated predators (Blanchard and Blanchard, 1989, Choi and Kim, 2010) to elicit fear responses. Responding to such predatory stimuli does not require previous experience or learning about the stimuli over multiple trials, and therefore fear toward these stimuli is considered to be genetically “pre-wired” (Öhman and Mineka, 2001). These ethologically relevant fears offer an evolutionary advantage over trial-and-error learning typical of associative fear learning, which can be both costly and time consuming for the organism (Pellman and Kim, 2016). Indeed, despite the prominence of associative fear learning models, it is debatable whether associative fear learning is the primary mechanism by which fear operates in nature (Bolles, 1970). Accordingly, the use of innate fear paradigms in fear research has been steadily increasing (Kim and Jung, 2018).

One of the most common predatory threats in rodents comes in the form of large aerial predators, such as owls (Andersson and Erlinge, 1977). On detection of overhead predators, rodents will display characteristic looming defense responses (LDRs), such as fleeing or freezing (Bolles, 1970), with the probability of either behavior prevailing being modulated by the perception of successful escape (Edut and Eilam, 2003, Fanselow, 1994, Yilmaz and Meister, 2013). Recently, the overhead, two dimensional (2D) “looming stimuli” developed by Yilmaz and Meister (2013) have been used to study LDRs in the laboratory setting (De Franceschi et al., 2016, Shang et al., 2018, Wallace et al., 2013, Wei et al., 2015). In these studies, rodents (usually mice) are placed in chambers commonly fitted with overhead monitors capable of producing visual stimuli. These stimuli are intended to simulate a rapidly approaching or distant aerial predator and come in the form of a rapidly expanding black disk or a sweeping black bar, respectively. Presentation of the looming stimuli transiently evokes freezing or fleeing, depending on whether the animal is distant from or nearby, respectively, an enclosed shelter providing a refuge (Yilmaz and Meister, 2013). The advantages of looming-induced fear paradigms are that the visual stimuli are simple to generate, precise, and highly controllable.

**Figure 1:**
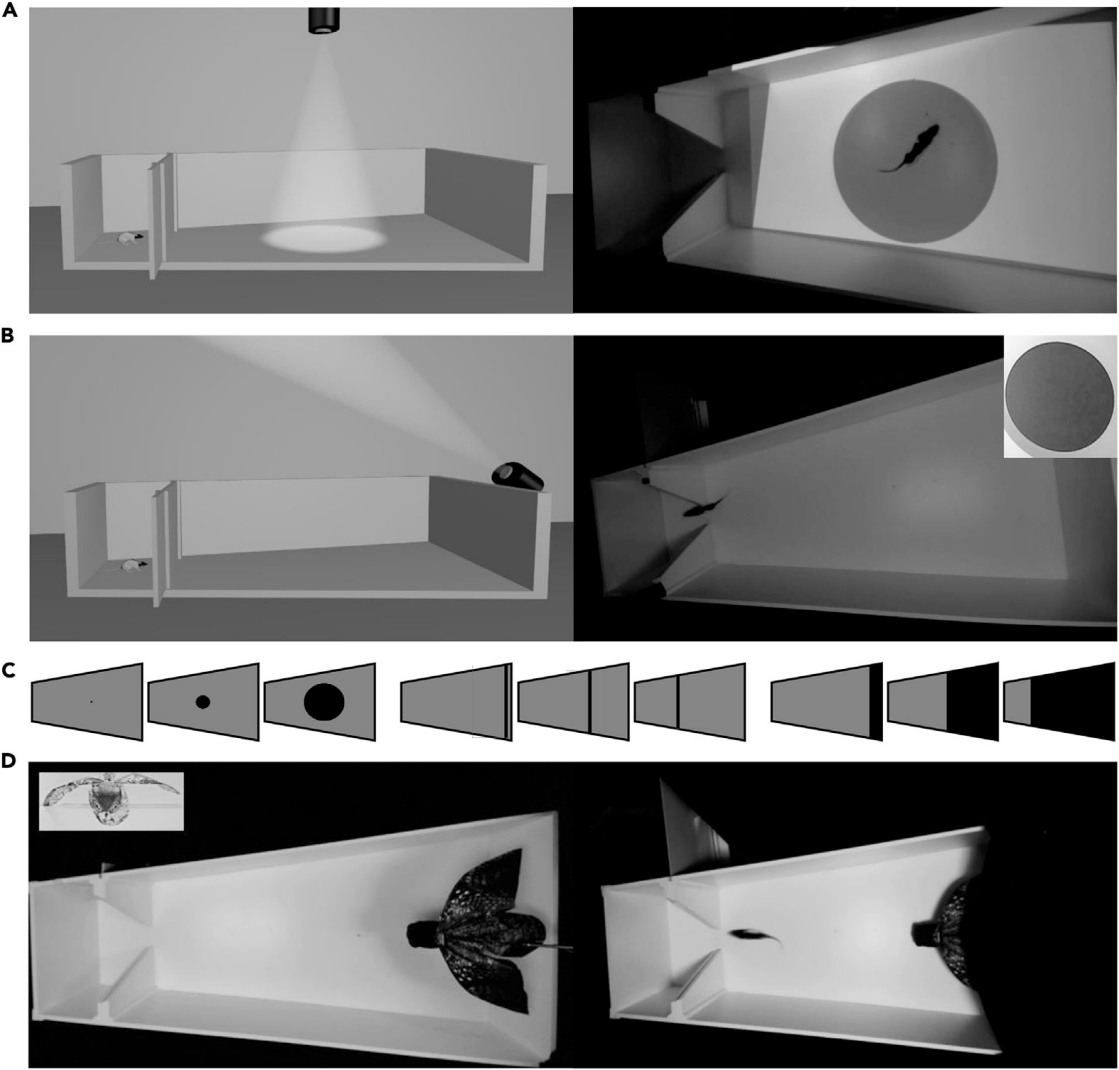
Experimental Apparatus and Looming Stimuli (A, left) 3D representation of nest (69 cm length × 58–66 cm width × 61 cm height) and foraging arena (208 cm length × 66–120 cm expanding width × 61 cm height) with floor looming projector orientation alongside (right) a still image of rat encountering expanding disc on the floor of the arena. (B, left) 3D representation of foraging arena with ceiling looming projector orientation alongside (right) a still image of a rat fleeing the overhead expanding disc stimuli. (C) Representations of each looming stimuli (expanding disc, sweeping bar, and large sweeping block) from beginning to end of the presentation and how they appear within the foraging arena. (D, left) Foraging arena owl in the prone position and no black-out curtain. Inset displays front-facing image of an owl. (D, right) Rat fleeing from activated owl (completely extended) back to the nest area. The arena was piped with masking white noise (72 dB at the aerial stimuli trigger zone). The sound associated with the activation of the owl briefly raised the total sound level to 75 dB.

Although previous studies have demonstrated that overhead 2D looming stimuli can evoke innate fear responding, the types of LDRs (freezing or fleeing to an enclosed nest) vary despite using similar stimuli and testing apparatus (De Franceschi et al., 2016, Yilmaz and Meister, 2013). Moreover, no looming stimuli study to date has utilized female rats; thus, any potential sex differences in rats are largely unknown. To further characterize and facilitate an understanding of innate fear responding to looming stimuli, the present experiment employed a more naturalistic, goal-directed risky-foraging task in male and female rats. Briefly, hunger-motivated animals are allowed to forage for food pellets in a large arena partitioned into two distinct zones: a safe, enclosed “nest area” and a risky, open “foraging area.” After a period of baseline foraging assessment, rats are challenged with presentations of the looming stimuli in the foraging area. Furthermore, in addition to using standard 2D looming stimuli projected above the animal (Yilmaz and Meister, 2013), there were projections of the stimuli onto the floor of the arena to examine whether animals display fear behavior toward shadows casted on the ground. We also employed a discrete, localizable three-dimensional (3D) looming stimulus, i.e., a life-like owl programmed to plunge toward the animal during the foraging task (Figure 1).

It was hypothesized that the 3D owl stimulus would be most effective in eliciting LDRs, as it more closely resembles an ethologically relevant threat compared with the 2D looming stimuli. To comprehensively test this hypothesis, separate groups of animals were challenged with the two stimuli types over multiple days of testing. Finally, we hypothesized that female rats would display greater innate fear to all looming stimuli and, consequently, be less successful in procuring pellets during stimuli testing. This is predicated on the previous literature showing that female rats react more defensively and engage in more risk-assessment behaviors following predator exposure (Blanchard et al., 1991, Pellman et al., 2017).

## Results

### Female Rats Initially Have Increased Foraging Times Than Male Rats

**Figure 2:**
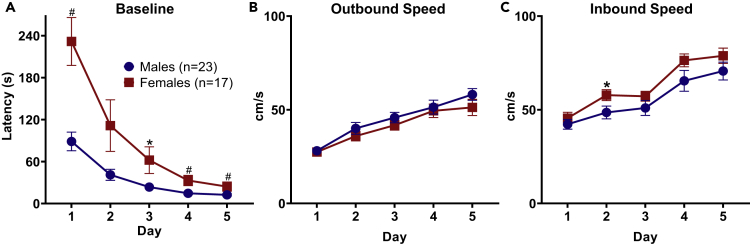
Average Latencies to Retrieve Pellet during Initial Baseline Session Days between Sexes Rats first engaged in a foraging task where food pellets were placed at increasing distances from the nest area, and no looming stimuli were presented. (A) Females took a significantly longer time foraging for food pellets than males on baseline session days 1, 3, 4, and 5. (B) There were no significant differences between sexes for outbound speed during baseline foraging days. (C) Inbound speed during baseline foraging where females were significantly faster on the second day. Data are represented as mean ± SEM. *p < 0.05, #p < 0.01.

Projector and owl encounter rats' data were pooled together as baseline foraging session methods were identical across experiments and no increase in latency was found from the projector being turned on, leading to baseline sample sizes of 20 male and 17 female rats. Rats were required to forage for food pellets at increasingly greater distances across the five baseline session days. Briefly, each baseline session day consisted of three trials in which a pellet was placed 25, 50, or 75 cm away from the nest (eventually increasing to 100 cm). As expected, the latencies to procure the pellet were longer on the first day since rats are venturing into a novel environment (Figure 2A). Additionally, the foraging apparatus expands in width as the rats move further away from the nest, adding a continuum of increasing danger to the task. A Mann-Whitney U test revealed female rats had significantly longer foraging times than male rats on all baseline session days, except for baseline session day 2 (Baseline 1: z = 3.535, p < 0.01; Baseline 2: z = 1.798, p > 0.05; Baseline 3: z = 2.424, p < 0.05; Baseline 4: z = 2.82, p < 0.01; Baseline 5: z = 2.851, p < 0.01). However, once the animals emerged from the nest, there were no significant differences between males and females in the outbound speed (distance/time) to the pellet location across any baseline days (Figure 2B; Mann-Whitney U, Baseline 1: z = −0.274, p > 0.05; Baseline 2: z = −0.958, p > 0.05; Baseline 3: z = −0.821, p > 0.05; Baseline 4: z = −0.123, p > 0.05; Baseline 5: z = −1.245, p > 0.05). Females on average had significantly higher inbound speeds (food pellet to the nest area) on the second day of baseline, but there were no significant differences on the following days (Figure 2C; Mann-Whitney U, Baseline 1: z = −0.821, p > 0.05; Baseline 2: z = −2.496, p < 0.05; Baseline 3: z = −1.724, p > 0.05; Baseline 4: z = −1.594, p > 0.05; Baseline 5: z = −1.246, p > 0.05). Although females took a significantly longer time foraging for pellets on the last baseline day, both sexes were quickly able to retrieve the pellet in under 25 s.

### 2D Floor Looming Elicited Weak Fear Responses

**Figure 3:**
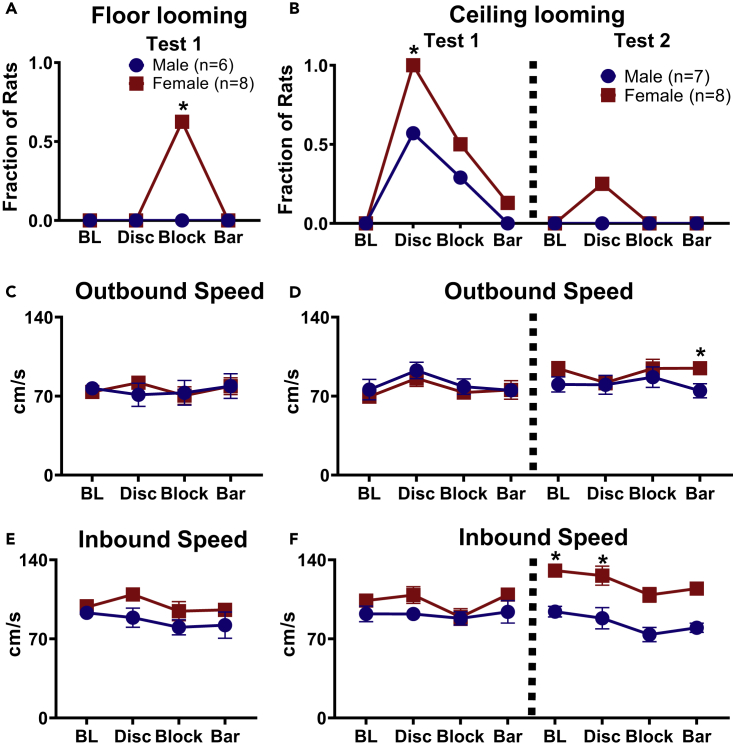
Higher Female Sensitivity to 2D Looming Stimuli (A) The fraction of male and female rats that fled from 2D floor-looming stimuli. (B) The fraction of animals that fled from 2D ceiling-looming stimuli. (C) Outbound speed during baseline and each projected stimuli. (D) There were no significant differences in outbound speed on the first day of testing. On the second day, females were on average significantly faster than males for the outbound speed during the Bar trial. (E) Inbound speed from the pellet location to the nest, the Block stimulus only includes females that fled to the nest during stimulus presentation (n = 5). Both sexes fled from the Expanding Disc in this condition. (F) There were no significant differences in inbound speed on the first day of projected stimuli presentations. Only the inbound speed data of males that fled from the Expanding Disc were used for analysis (n = 4). Females had significantly faster inbound speeds on the second day of projector testing during the Baseline and Disc trials. On the second testing day of Ceiling Looming most males consumed the food pellet outside the nest area, so inbound speed for only two males could be calculated. Data are represented as mean ± SEM. *p < 0.05.

Foraging rats were exposed to a 2D stimulus, an expanding disc, a large sweeping block, or a sweeping bar, projected onto the floor of the arena (Figure 1A). None of the male rats exhibited LDRs to floor looming stimuli, whereas 5 of 8 females fled from the block stimulus but not the other visual stimuli (Figure 3A). A chi-square test for independence found that females had a significantly higher fear response than males to the floor looming block (*χ*^2^[1, *n* = 14] = 5.833, p = 0.016). This fleeing response quickly habituated following repeated presentations. The outbound and inbound speeds were compared between males and females with no significance found (Figures 3C and 3E; Outbound: Mann-Whitney U, Baseline, z = −0.645, p > 0.05; Disc, z = −0.387, p > 0.05; Block, z = 0, p > 0.05; Bar, z = −0.214, p > 0.05. Inbound: Baseline, z = −0.665, p > 0.05; Disc, z = −1.717, p > 0.05; Block, z = −1.278, p > 0.05; Bar, z = −1.393, p > 0.05). For the inbound speed during Block stimulus trials, only the speeds of females that fled from the stimulus were included in the analysis (n = 5). Freezing behavior (i.e., no movement apart from respiration for ≥2 s) was never observed in males and females during the floor looming test.

With respect to looming stimuli presentation, the first cohort of rats tested was presented with the large block stimulus first, the expanding disc second, and the sweeping bar third, whereas the second cohort was exposed to the expanding disc first, the sweeping bar second, and the large block third. The pellet was also placed further from the nest area (175 cm). Among the females, there was no order-effect found for stimuli presentation, with females in either cohort responding to the large block regardless of its presentation order. None of the animals underwent both the floor looming and ceiling looming conditions. It is possible, however, that an animal that did not show a fear response to the floor looming stimuli would exhibit fear toward the ceiling looming stimuli if tested consecutively. No significant differences were found between sexes for latencies to procure the pellet for each stimulus using a Mann-Whitney U test (expanding disc: z = 1.68, p > 0.05; large block: z = 0.389, p > 0.05; sweeping bar: z = 0.969, p > 0.05).

### 2D Ceiling Looming Elicited Moderate Fear Responses

**Figure 4:**
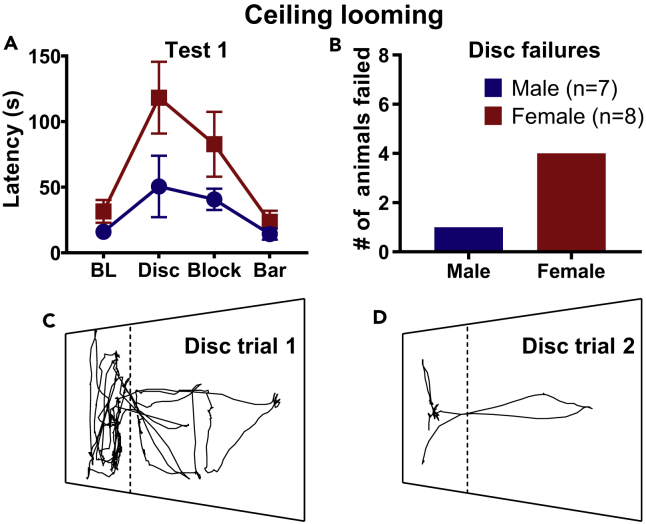
Foraging Latencies to Ceiling Looming Stimuli (A) Group average latencies ±SEM to retrieve food pellet. (B) A number of rats who failed to retrieve the pellet on Expanding Disc trial (did not retrieve pellet within 180 s). (C) Representative position plots of a female rat that failed to retrieve a pellet on the first day of Expanding Disc exposure. (D) Position plot from the same rat demonstrating rapid habituation to Expanding Disc during the second day of expanding disc exposure.

The floor looming condition produced very weak fear in few females and none in males, whereas the visual stimuli projected onto the ceiling above the foraging arena (“Ceiling looming” condition; Figure 3B) were moderately effective in evoking LDRs (Video S1). Specifically, a chi-square test for independence revealed that females (8/8) responded significantly more than males (4/7) to the ceiling looming disc (*χ*^2^[1, *n* = 15] = 4.29, p = 0.038). The sweeping block stimulus elicited fewer fleeing responses in both sexes (4/8 females; 2/7 males), whereas almost none of the animals (1/7 females; 0/8 males) fled in response to the overhead, sweeping bar stimulus. Once again, none of the male and female rats displayed freezing in the arena when looming stimuli were presented and quickly habituated to the stimuli (Figure 4B-4D). Hence, our sweeping stimuli results in rats contrast with a previous study conducted in mice that reported freezing responses to a similar stimulus (De Franceschi et al., 2016).

Video S1. Ceiling Looming Expanding Disc, Related to Figure 3 An example rat triggering expanding disc stimuli, which causes a fleeing response to the nest. A mirror mounted above the end of the arena shows the projected looming stimulus on the ceiling.

Latencies to procure the pellet mirrored the fleeing response data, such that the highest latencies occurred with the expanding disc, followed by the block and bar stimuli, respectively (Figure 4A). Each trial lasted a maximum of 180 s. Half of the female rats and 1 male rat reached this cutoff before procuring the pellet (Figure 3B). There were no significant differences in outbound speed in either testing day, except for females having significantly higher speeds during the Bar trial on testing day 2 (Figure 3D; Mann-Whitney U, Test 1: Baseline: z = −0.579, p > 0.05 expanding disc: z = −0.340, p > 0.05; large block: z = −0.694, p > 0.05; sweeping bar: z = −0.463, p > 0.05. Test 2: Baseline: z = −1.62, p > 0.05 expanding disc: z = −0.463, p > 0.05; large block: z = −0.688, p > 0.05; sweeping bar: z = −2.199, p < 0.05). There were no significant changes in inbound speed following stimulus exposure (Figure 3F), and only the rats that fled from the stimulus (as mentioned earlier) were included in the analysis (Mann-Whitney U, Test 1: Baseline: z = −1.678, p > 0.05 expanding disc: z = −1.189, p > 0.05; large block: z = −0.387, p > 0.05; sweeping bar: z = −1.254, p > 0.05). On the second day of projector testing most males (n = 6) consumed the food pellets in the foraging area so no inbound speed to the nest was able to be calculated, resulting in a low n-size across all trials (Figure 3F; Mann-Whitney U, Test 2: Baseline: z = −2.646, p < 0.05 expanding disc: z = −2.089, p < 0.05; large block: z = −2.049, p > 0.05; sweeping bar: z = −2.049, p > 0.05). Interestingly, females exhibited a large increase in inbound speed during this day of testing. In general, there were trends of female rats exhibiting higher latencies to procure the pellet than male rats during the stimulus trials (Mann-Whitney U, expanding disc: z = 1.532, p > 0.05; large block: z = 1.101, p > 0.05; sweeping bar: z = 0.928, p > 0.05).

### 3D Predatory Threat Evokes a Stronger Fear Response in Female Than Male Rats

**Figure 5:**
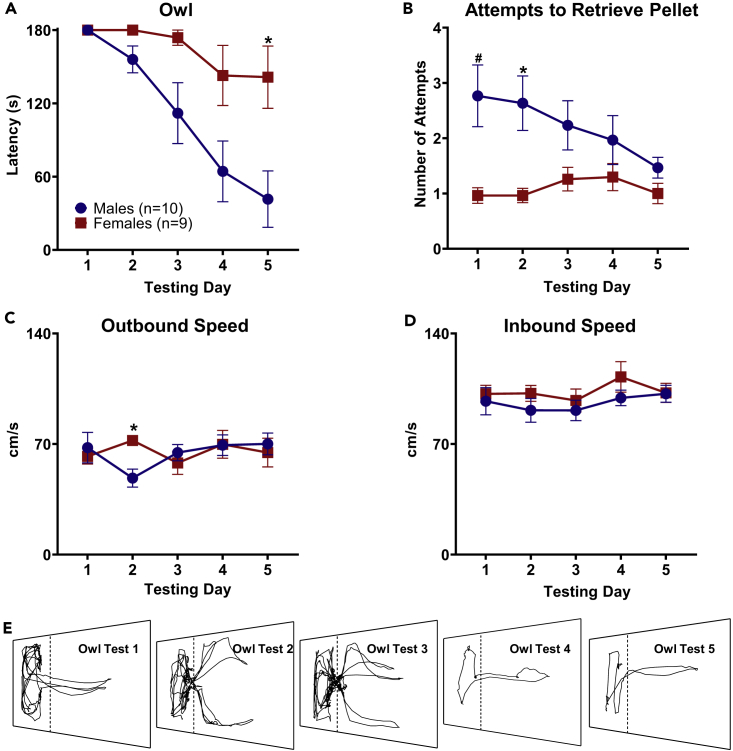
Female Rats Exhibited Stronger Fear to the Owl Than Male Rats Across the Testing Days (A) Male and female rats average group latencies ±SEM to retrieve the pellet under aerial predator threat. Male rats habituated to the owl faster than females. (B) Average attempts made to procure the food pellet across owl exposure days. Male rats made significantly more attempts than females to reach the food pellet, leading to more exposure to the predator early on in testing. *p < 0.05, #p < 0.01. (C) Outbound speed during owl exposure testing days. Females were significantly faster outbound on day 2. (D) There are no significant differences between males and females speed during inbound responses to the owl. (E) Representative position plots of male rat across 5 days of Owl exposure.

Rats foraging for pellets placed 100 cm away from the nest encountered a model owl that emerged from behind a blackout curtain. Two subsequent trials each testing day had the pellet placed at 75 and 50 cm away from the nest, totaling to three (180 s) trials per day. The latency to procure the pellet and number of attempts to acquire the pellet (i.e., the number of owl activations) were averaged each testing day. Initially, both male and female rats failed to procure the pellet under threat of the owl (Figure 5A, Video S2). Across days, however, male rats habituated to the owl faster than female rats, with a significant difference in latency on day 5 (Mann-Whitney U test, z = 2.331, p < 0.05; Figure 5E). Notably, it took 19 days for all female rats to successfully procure the pellet during the three baseline and three owl exposure trials given per test day (Figure S1). During the first 2 days of owl exposure, male rats also triggered the owl significantly more (Figure 5B; Mann-Whitney U test, Owl day 1: z = 2.684, p < 0.01; Owl day 2: z = 2.199, p < 0.05), signifying more attempts were made to retrieve the food pellet. There is a noticeable decrease in outbound speed in male rats on day 2 following owl exposure (Figure 5C), and on the same testing day females had significantly higher outbound speeds (Mann-Whitney U test, Day 2 Outbound Speed: z = −3.022, p < 0.05). There were no other significant differences in outbound or inbound speed (Figure 5D; Mann-Whitney U test, Outbound, Day 1: z = −0.408, p > 0.05, Day 3: z = −0.653, p > 0.05, Day 4: z = −0.041, p > 0.05, Day 5: z = −0.449, p > 0.05. Inbound, Day 1: z = −0.082, p > 0.05, Day 2: z = −0.735, p > 0.05, Day 3: z = −0.123, p > 0.05, Day 4: z = −0.776, p > 0.05, Day 5: z = −0.204, p > 0.05).

### Contextual Fear from 3D Predatory Threat Encounters

**Figure 6:**
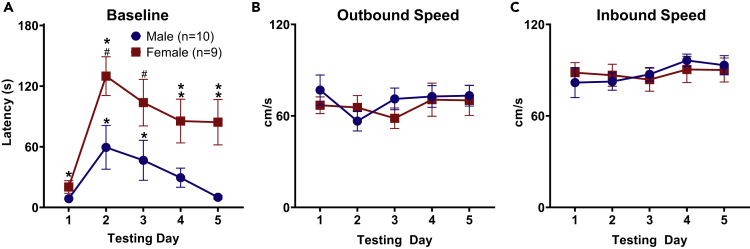
Sex Differences in Contextual Fear from Owl Encounters (A) Group average latencies + SEM to procure the food pellet during baseline trials performed on each testing day before the Owl encounter trials. Male and female rats both exhibited significantly longer baseline foraging times the day following the predator encounter when compared to the first day of predator exposure. Female rats took a significantly longer time than males to procure the pellet on days 1, 2, 4, and 5. *p<0.05, #p<0.01. (B) There are no significant differences between males and females during outbound responses to the owl. Data are represented as mean ± SEM. (C) There are no significant differences between males and females during inbound responses to the owl. Data are represented as mean ± SEM.

Each owl testing day began with three baseline trials (pellet placed at 100 cm) to measure any contextual fear memory that may have transpired from encounters with the owl the previous day. Both male and female rats' baseline trial latencies significantly increased following the first day of owl exposure (Figure 6A; related samples Wilcoxon signed ranks test, Males: BL1-BL2, z = 2.395, p < 0.05; BL1-BL3, z = 2.296, p < 0.05. Females: BL1-BL2, z = 2.666, p < 0.01; BL1-BL3, z = 2.666, p < 0.01; BL1-BL4, z = 2.521, p < 0.05; BL1-BL5, z = 2.134, p < 0.05). Females, however, exhibited a stronger contextual fear response than males during owl testing days 1, 2, 4, and 5 baseline trials (Mann-Whitney U test, BL1: z = 3.535, p < 0.01; BL2: z = 1.798, p > 0.05; BL3: z = 2.424, p < 0.05; BL4: z = 2.82, p < 0.01; BL5: z = 2.851, p < 0.01). There were no significant differences in outbound or inbound speed during baseline trials on owl testing days (Figures 6B and 6C; Mann-Whitney U test, Outbound, Day 1: z = −0.899, p > 0.05, Day 2: z = −0.613, p > 0.05, Day 3: z = −1.551, p > 0.05, Day 4: z = −0.683, p > 0.05, Day 5: z = −0.898, p > 0.05. Inbound, Day 1: z = −0.613, p > 0.05, Day 2: z = −0, p > 0.05, Day 3: z = −0.41, p > 0.05, Day 4: z = −1.103, p > 0.05, Day 5: z = −0.082, p > 0.05).

## Discussion

Before looming stimuli testing, evidence of sex differences was apparent during the baseline foraging sessions. Female rats were more reluctant to enter the larger, open foraging area to obtain the food pellet, preferring to remain in the safe, enclosed nest area. This is especially evident on the first baseline day. These baseline data are in accordance with reports that female rats spend less time out of cover and are more hesitant to consume food in a novel environment (Carrier and Kabbaj, 2013, Jolles et al., 2015, Turner and Burne, 2014).

More female than male rats fled from the ceiling looming expanding disc on the first day of testing. The ceiling looming expanding disc most accurately simulates a rapidly descending predator among the 2D stimuli, whereas the ceiling bar stimulus is intended to resemble an approaching “cruising” predator (De Franceschi et al., 2016, Yilmaz and Meister, 2013). That more females than males displayed LDRs specifically to the ceiling looming disc suggests that female rats show exaggerated fear toward imminent predation threat and not just more defensive/risk-assessment behavior following predator exposure as previously reported (Blanchard et al., 1991). Notwithstanding, female rats showed greater hesitancy to leave the safe nest area following stimulus presentation during testing, reflected in their increased latencies and failed attempts to procure the pellet.

Previous research has shown that Long-Evans female rats perform better than males in an object recognition task in which visual acuity was measured (Seymoure and Juraska, 1997). Thus, it is possible that the observed sex differences in the 2D looming experiments may be explained by a poorer vision in male rats. However, differential fear responding was evident when stimuli were projected downward onto the arena, which minimizes visual acuity differences. Specifically, more females than males fled from the floor looming block stimulus.

The block stimulus encompassed a greater area when projected onto the arena than when projected upward onto the smaller projector screen. Thus, during the floor looming condition, when the shapes of the stimuli are largely indecipherable, the block stimulus may be perceived as higher in intensity and therefore in aversiveness compared with the floor disc and bar stimuli, which do not cover the entirety of the arena. One way to possibly eliminate this difference is by lowering the ceiling projector screen so that the stimuli appear more similar in size between conditions. The floor looming block stimulus also produces a stronger change in arena brightness. Insofar as male mice are concerned, rapid changes in brightness alone do not elicit an innate fear response (Yilmaz and Meister, 2013), which is consistent with our male rat results. Nonetheless, such a large and fast change in illumination may be perceived as aversive in female rats. Finally, the absence of LDRs in males and females to the floor projected expanding disc may also be indicative of the visual system's need to perceive a rapidly approaching predator from above to initiate an LDR and not merely the shadow cast by the predator on the floor beneath the rat. Yilmaz and Meister (2013) have also shown in mice that only when presented above does the expanding disc trigger a flight response.

When animals were confronted with the 3D looming stimulus, a plastic owl “predator,” there was no difference in LDRs on the first day of testing, and all animals failed to obtain the pellet. However, differences emerged over subsequent test days. Starting with the second test day, male rats' latencies began to rapidly decrease with each subsequent test. Female rats' latencies only began to decrease starting the third day of testing but ultimately remained high for the remainder of the testing schedule. Also pronounced is the difference in baseline trial latencies following the first test day. Female rats' baseline trial latencies remained significantly higher than in male rats consistently across the remaining test days, evidence of a stronger contextual fear memory formed after initial owl exposure and greater resistance to extinction. Such findings conflict with data generated from context fear conditioning studies, which show that male rats display greater levels of fear when freezing is used as the index of fear (Colon et al., 2018, Graham et al., 2009, Maren et al., 1994) and that females are quicker to return to areas previously associated with footshock (Beatty et al., 2013, Heinsbroek et al., 1988, Van Oyen et al., 1979). Notably, none of the animals displayed freezing behavior to either the 2D or 3D looming stimuli in the foraging area.

Few significant changes in outbound or inbound speed were observed throughout baseline and testing sessions. Those that were significant did not correlate with a fear response or a rapid return to the nest area and might be an effect of the animals having completed many trials. As stated earlier, most male rats began consuming the food pellets in the foraging area during the second day of projector testing, indicating low fear to that space, even though it is a bright-light condition. That the female rats had significantly higher outbound speeds than male rats on the second day of owl exposure is in agreement with past research showing that female rats will explore an area previously associated with predator odors faster than male rats but show heightened fear to the odor itself (Jolles et al., 2015). Female rats have an increase in outbound speed over male rats immediately before the owl is triggered, but male rats habituate to the direct predator encounter at a substantially faster rate. As outbound and inbound speeds were not reliably different across test days, sex differences in latency to procure the pellet were due to increased hesitancy to approach the pellet as well as number of attempts during owl encounters.

Factors relating to our foraging paradigm, chamber design, and species tested may explain the discrepant findings discussed earlier. In our paradigm, the animals are engaged in goal-oriented, purposive behavior (Tolman, 1932) in a large arena, which effectively expands the animal's behavioral repertoire. This paradigm was also designed to simulate an ethologically relevant “approach food–avoid predator” conflict situation, contrasting with the paradigms in which animals are arbitrarily placed into small chambers that restrict movement and behavioral capacity. However, the animals in the present study also had extensive experience in the arena before testing and undoubtedly had thoroughly mapped the locations of the safe (nest area) versus dangerous (foraging area) zones (Kim et al., 2015). These factors perhaps explain why we observed only fleeing LDRs in both 2D and 3D looming experiments. In regards specifically to our 2D looming stimuli results, the fact that we used rats instead of mice may explain why none of the animals froze to the expanding disc, despite being at a considerable distance from the nest area during stimulus presentation. Although physically similar, rats and mice show considerable evolutionary divergence (Gibbs et al., 2004) and are thus not entirely interchangeable in behavioral experiments.

Lastly, the predator stimuli used were also made more realistic in our design, such that 2D stimuli were presented above the animal at more plausible heights (2.19 m) instead of being presented 30 cm above the animal, as in contemporary looming studies (De Franceschi et al., 2016, Wallace et al., 2013, Wei et al., 2015, Yilmaz and Meister, 2013). The 3D looming stimulus also realistically resembled an owl. While the 3D looming stimulus uniquely produced sound (a 3 dB increase in the background of 72 dB white noise) and potentially caused an air breeze upon trigger—potential confounds—the owl likely represents a more intense predator stimulus than the 2D stimuli, which can support contextual fear conditioning (Blanchard et al., 2003). In fact, most animals fully habituate to the 2D stimuli by test day 2, suggesting that they are relatively weak danger stimuli. Indeed, one other study performed 2 days of testing with an overhead expanding disc and likewise found a dramatic reduction in fear responses by the second day of testing (Wei et al., 2015).

An evolutionary-developmental theory may explain in a more general sense the observed sex differences in risky-foraging behavior. As male and female mammals develop, divergent behaviors are predicted to emerge as a result of differential reproductive and survival goals (Ellis et al., 2009, Kodric-Brown and Brown, 1987, West-Eberhard, 2014). Males typically devote more effort toward reproduction, whereas females typically devote more effort toward offspring development (i.e., “parenting”); thus, risk-taking may be more beneficial for males in that it can increase the chances of mate access, whereas risk-aversion in female rats helps ensure stable parenting of offspring (Jolles et al., 2015, Kodric-Brown and Brown, 1987, Steinberg, 2008). However, the possibility that food restriction, implemented in our study to motivate the animals to engage in the foraging task, in part accounted for the observed sex differences cannot be excluded.

In conclusion, both 2D and 3D looming stimuli can elicit LDRs in male and female rats, albeit differentially. 2D looming stimuli are most effective in female rats, who reliably respond to the ceiling looming expanding disc on the first day of testing. Males, on the other hand, responded to the expanding disc at lower rates. Both sexes exhibit robust fear responding to a 3D owl stimulus, which can support contextual fear conditioning. However, female rats consistently show greater, longer-lasting fear across the owl test sessions. Ultimately, these data reflect the notion that female rats are more sensitive to aerial predation threat and respond more defensively following predator exposure. Notably, in contrast to fear conditioning studies that reported stronger contextual freezing in male rats than females rats (Colon et al., 2018, Graham et al., 2009, Maren et al., 1994), our ecologically relevant foraging with looming threat paradigm, where female rats show significantly greater and enduring fear behavior than male rats, may have translational relevance as the observed sex differences parallel human conditions where women have a higher incidence of anxiety and posttraumatic stress disorders than men (Olff, 2017). Future experiments utilizing our naturalistic looming paradigm may seek to identify the neural circuits involved in generating the general LDR seen in both sexes following 2D and 3D stimuli, as well elucidate whether potential differences in anatomy and/or function of those circuits contributes to the observed sex differences.

### Limitations of the Study

All experiments were performed on the Long-Evans strain of rat and purchased from Charles-Rivers Laboratories. Future experiments could include different rat strains from other vendors to ensure the sex differences we have found are ubiquitous among rats. The rapid habituation of fear in both sexes and lower male response rate to the 2D looming stimuli might prove impractical for those planning on using more advanced techniques like single-unit recordings and calcium imaging, which require several testing trials. The 3D owl predator stimulus overcomes this limitation, but requires a much larger space to simulate naturalistic “approach food–avoid predator” conflict situations.

## Methods

All methods can be found in the accompanying Transparent Methods supplemental file.

## Supplemental Information

Document S1. Transparent Methods and Figure S1

Video S2. Rat Fleeing from 3D Predator, Related to Figure 5 A rat attempting to forage for food while encountering the owl predator.
