# Supplementary material for: Sex Differences in Foraging Rats to Naturalistic Aerial Predator Stimuli
Source: iScience. 2019 Jun 11;16:442–52. doi: 10.1016/j.isci.2019.06.011 (PMC6593150; doi:10.1016/j.isci.2019.06.011)
Supplement: Document S1. Transparent Methods and Figure S1 [file mmc1.pdf]

ISCI, Volume *16*

Supplemental Information

Sex Differences in Foraging Rats to Naturalistic Aerial Predator Stimuli

Peter R. Zambetti, Bryan P. Schuessler, and Jeansok J. Kim

Supplemental Information

Figure S1. Average latencies across owl testing days. Related to Figure 5.

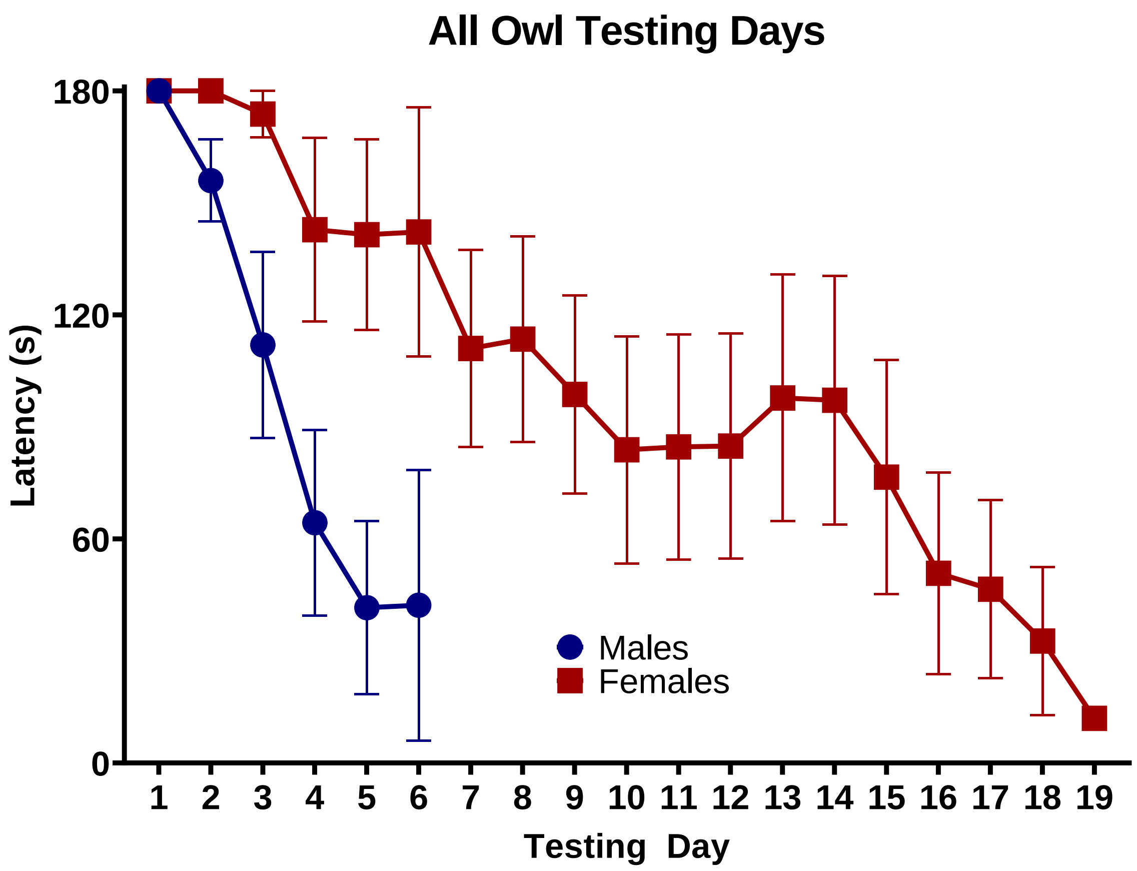

Supplemental Figure Legends

Supplemental Figure 1. Average latencies across owl testing days. Related to Figure 5. Mean group latencies ± SEM for male and female rats. For females, n=9, n=7 and n= 6 rats for testing days 1-12, 13-17 and 18-19, respectively. For males, n=10 and n=4 for testing days 1-5 and day 6, respectively.

Supplemental Video 1. Ceiling looming expanding disc. Related to Figure 3. An example rat triggering expanding disc stimuli, which causes a fleeing response to the nest. A mirror mounted above the end of the arena shows the projected looming stimulus on the ceiling.

Supplemental Video 2. Rat fleeing from 3D predator. Related to Figure 5 A rat attempting to forage for food while encountering the Owl predator.

Transparent Methods

Subjects
A total of 40 rats (23 males and 17 females; nulliparous; 3-4 months old) were ordered from Charles-Rivers Laboratories in small cohorts (n=4 for each sex) and tested independently. Once transported, animals were individually housed in a climate-controlled vivarium (accredited by the Association for Assessment and Accreditation of Laboratory Animal Care) and maintained on a reverse 12-h light/dark cycle (lights on at 7:00 PM). After 5-7 days, animals were placed on a standard food deprivation schedule (with *ad libitum* access to water) to gradually reach and maintain 85% of their normal weight. All experiments were conducted during the dark phase of the cycle and in compliance with the University of Washington Institutional Animal Care and Use Committee guidelines.

Foraging Apparatus
A custom-built foraging arena consisted of a nest (69 cm L x 58-66 cm W x 61 cm H) that opened via a sliding gate to reveal a large foraging area (208 cm L x 66-120 cm W x 61 cm H) where food pellets were placed and where rats encountered 2-D and 3-D looming stimuli. The testing room was kept under red light (11 lux foraging area, 2 lux nest area) with constant white noise (72 dB) playing in the background. The ANY-maze software and AMi interface system (Stoelting) connected to a PC tracked the animal's position in the arena, via a ceiling mounted camera, and triggered the looming stimuli.

Floor looming. A drop-ceiling projector mount (Amer) was used to position the projector (Epson EX7240) above and perpendicular to the foraging zone so that nearly the entire arena (except the nest) was covered by the projected image (85 lux).

Ceiling looming. The projector was mounted at the end of the foraging arena and directed upwards to a white insulation foam (244 x 122 cm) screen suspended 2.19 m above the foraging arena (52 lux).

Owl. A life-like model owl (84 cm wingspan) was mounted to a pneumatic air cylinder (Bimba, 92 cm) and positioned at a 45° angle above the arena (76 cm) at the opposite end from the nest. A bidirectional double-solenoid air-valve (IMI Norgren) placed outside of the arena was used to activate the air cylinder, which generated an increase to 75 dB (from 72 dB) in the foraging arena when the valve switched position. Exhaust dampeners (McMaster-Carr) were attached to the solenoid valve ports to lessen noise. While in the retracted position, the owl was hidden from the view of the animal by a black curtain spanning the width of the apparatus. When fully extended (at the rate of 46 cm/s), the bottom of the owl was 43 cm above the arena floor at the long pellet location (100 cm from nest). The owl was triggered when the animal entered predetermined distances of 75, 50, and 25 cm from the nest for long, medium, and short pellet location tests, respectively.

Behavioral Procedures
All animals underwent habituation and baseline foraging sessions prior to floor looming, ceiling looming or owl encounter tests on consecutive days. The ANY-maze tracking data (sampled at 60 Hz) were used offline to quantify (i) freezing, defined as no movement apart from respiration (Blanchard and Blanchard, 1969) for at least 2 sec, (ii) outbound foraging speed (the time from exiting the nest to either reaching the pellet or entering the looming stimuli trigger zone), (iii) inbound speed (the time from either procuring the pellet or triggering the looming stimuli to

returning to nest) and (iv) latency to procure food pellet (time it takes for rat to retrieve pellet and begin consumption). Fleeing/escape behavior was defined as the animal running back to the nest without securing the pellet.

Habituation. Rats were placed in the nest area for 30 min/day for two days with 20 food pellets (0.5 g, Bio-Serv dustless precision pellets, #F0171) to acclimate to the experimental setting and food source, and to also associate the nest area with feeding.

Baseline sessions. Each day rats were placed in the nest area with 2 food pellets. Once the pellets were eaten, the nest door to the foraging area opened with a pellet placed 25 cm away. The latency to retrieve the pellet and return to the nest area for consumption was measured, after which the door was closed. This process repeated with the pellet placed 50 and 75 cm away from the nest opening. Across the subsequent 4 days, the pellet gradually moved further away from the nest until it reached 100 cm from the nest opening. From the 3rd day of baseline through the end of testing, the projector was on to habituate rats to projector generated background light and sound. The owl encountering rats remained in red light throughout the entire experiment.

Floor looming testing. On the test day, animals initially underwent 3 pre-looming baseline trials with 100 cm pellet placement. Afterwards, the pellet was moved 100-175 cm from the nest for looming tests. When the rat was within ~25 cm of the pellet, the experimenter remotely activated black expanding disk, sweeping bar and sweeping block stimuli (counterbalanced order). Specifically, the expanding disk started at the center of the floor with a diameter of 0.635 cm and extended to 66 cm (at a rate of 59.43 cm/s) encompassing nearly the entire foraging arena; the sweeping bar was a narrow rectangle that moved at 61.21 cm/s to the nest; and the large sweeping block started at the edge and filled the entire foraging area at 61.21 cm/s. During each stimulus test (3 minutes), as the animal entered the trigger zone, the looming stimulus was triggered five times in rapid succession. The visual stimuli were created and converted into executable files using Python packages TKinter/Turtle/PyInstaller, and inserted into an ANY-maze procedural file.

Ceiling looming testing. Animals underwent the same procedure as described above, except the looming stimuli projected on the ceiling.

Owl encounter testing. Each testing day began with rats being placed into the nest with 2 food pellets and then undergoing 3 pre-owl baseline trials with the pellet placed 100 cm away from the nest. Afterwards, each time the rat was within ~25 cm of the pellet, the owl automatically plunged towards the rat, and after one second retracted to its original position behind the blackout curtain. If the animal was unable to procure the pellet within 3 minutes, on subsequent trials, the pellet was moved closer to the nest (75 and 50 cm away) and the animals were again given 3 minutes to retrieve the pellet. Daily testing continued until all rats successfully procured the pellet while facing the owl. Four of the 8 female rats previously underwent 2D floor looming stimuli; however, there were no differences in behavior between 2D pre-exposed and naïve rats to the owl.

Data Analyses
Statistical analyses were performed using SPSS (IBM, version 19), and graphs were created using GraphPad (Prism, version 8). Because the Levene’s test for normality showed significance, nonparametric tests were used to analyze data.

Baseline sessions. To compare initial baseline latencies to procure the pellet, Mann-Whitney U tests were used to compare male and female rats.
2-D looming testing. Chi-square tests for independence were applied to both floor and ceiling looming data to compare the escape response frequency between males and females. A Mann-Whitney U test was also used to compare the latency to procure the food pellet between sexes for each 2D looming type.

Owl testing. A Mann-Whitney U test was used to determine sex differences in the foraging latencies and speed, and the number of attempts to procure the pellet across the baseline and owl encounter trials.

Supplemental References

Blanchard R.J. and Blanchard D.C. (1969). Crouching as an index of fear. J Comp Physiol Psychol 67, 370–375.
